# Seasonal variations of intensity of avian malaria infection in the Thousand Island Lake System, China

**DOI:** 10.1186/s13071-023-05848-4

**Published:** 2023-07-04

**Authors:** Yuxiao Han, Olof Hellgren, Qiang Wu, Juan Liu, Tinghao Jin, Staffan Bensch, Ping Ding

**Affiliations:** 1 MOE Key Laboratory of Biosystems Homeostasis & Protection, College of Life Sciences, Zhejiang University, Zhejiang, China; 2 Molecular Ecology and Evolution Lab, Department of Biology, Lund University, Lund, Sweden

**Keywords:** Bird migration, Infection intensity, Hemosporidia, Disease dynamics, Avian malaria

## Abstract

**Background:**

Migratory birds play an important part in the spread of parasites, with more or less impact on resident birds. Previous studies focus on the prevalence of parasites, but changes in infection intensity over time have rarely been studied. As infection intensity can be quantified by qPCR, we measured infection intensity during different seasons, which is important for our understanding of parasite transmission mechanisms.

**Methods:**

Wild birds were captured at the Thousand Island Lake with mist nets and tested for avian hemosporidiosis infections using nested PCR. Parasites were identified using the MalAvi database. Then, we used qPCR to quantify the infection intensity. We analyzed the monthly trends of intensity for all species and for different migratory status, parasite genera and sexes.

**Results:**

Of 1101 individuals, 407 were infected (37.0%) of which 95 were newly identified and mainly from the genus *Leucocytozoon*. The total intensity trend shows peaks at the start of summer, during the breeding season of hosts and during the over-winter season. Different parasite genera show different monthly trends. *Plasmodium* causes high prevalence and infection intensity of winter visitors. Female hosts show significant seasonal trends of infection intensity.

**Conclusions:**

The seasonal changes of infection intensity is consistent with the prevalence. Peaks occur early and during the breeding season and then there is a downward trend. Spring relapses and avian immunity are possible reasons that could explain this phenomenon. In our study, winter visitors have a higher prevalence and infection intensity, but they rarely share parasites with resident birds. This shows that they were infected with *Plasmodium* during their departure or migration and rarely transmit the disease to resident birds. The different infection patterns of different parasite species may be due to vectors or other ecological properties.

**Graphical abstract:**

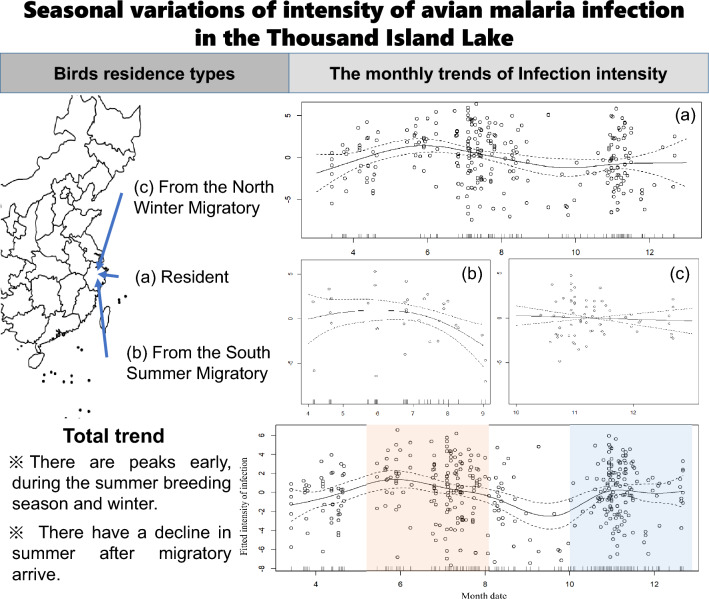

**Supplementary Information:**

The online version contains supplementary material available at 10.1186/s13071-023-05848-4.

## Background

Emerging infectious diseases are a serious challenge for human health [1], with infectious diseases killing millions of people every year [2] and threatening the survival of wild animals [3]. Avian hemosporidiosis is a global infectious disease widely prevalent among wild birds and caused by blood parasites [4] of the genera *Plasmodium*, *Haemoproteus* and *Leucocytozoon* [5]. Recently, increasing research has been done on avian hemosporidians. Hemosporidians multiply as haploid clones in the cells of the fixed tissues of avian hosts and undergo the sexual process in vectors [6]. Some *Plasmodium* lineages, like SGS1, can infect at least hundreds of birds species [7], and the same bird species can also be infected by different parasites [8]. The interaction between hosts and parasites is a complex system, which includes individuals [9], species [10], communities [11], potential vectors and other different organizational scales [12, 13]. Due to the complexity of the avian hemosporidian host-parasite system together with the relatively ease by which it can be studied in the wild, avian hemosporidiosis has become a model for studies on disease dynamics in the wild [14].

Previous studies have found seasonal variations in infectious diseases [15, 16]. The mechanism leading to this change is worth exploring. Vectors [6], host migration [17], flocking behavior and habitat [18] are all factors that lead to changes in prevalence. Due to host body mass and conditions, host migration or insect vectors [19–21], the prevalence of avian hemosporidians shows seasonal and annual variations [22, 23]. A model from Beaudoin et al. found a hypothetical model predicting that a peak in malaria prevalence is supposed to occur in late summer and autumn [24]. Prevalence also increases when the host's immune system is weakened [25], so birds are usually infected when they are fledglings [26]. This leads to an increased prevalence during the breeding season. On the other hand, there are different host preferences and infection specificities within the genera *Plasmodium*, *Haemoproteus* and *Leucocytozoon* [27], which could further lead to different seasonal trends among different genera.

It is a well-known fact that migratory animals play an important part in the spread of parasites and diseases [28, 29] as they can travel great distances while spreading parasites [30–32], especially during wintering and breeding seasons [33]. For example, 4 to 39 million exotic neotropical ticks are spread to the USA annually by migratory songbirds [34], and different parasite transmission strategies or coevolution between the host and avian malaria parasite lineages cause the different prevalences found in Garden warblers (*Sylvia borin*) [35]. In Barn swallows (*Hirundo rustica*), the prevalence of Africa-transmitted *Plasmodium* lineages was significantly higher in individuals overwintering in the moist habitats of Western Central Africa [36]. Moreover, the Thousand Island Lake (TIL), located in the south of the Asian continent, has both summer and winter avian visitors [37], and also some passing birds, so it has a different migration pattern from the previous main research in Europe and North America. Thus, it gives us a chance to learn how the different migratory birds might affect the parasite communities in local resident birds.

However, most studies focused on the effect of residency types on prevalence, but infection intensity has rarely been considered. The intensity of blood parasites might not only have a link with how they affect the hosts (although the effects might vary between different host-parasite combinations) but also with the dynamics of transmission. Some research shows that specialist parasites are well adapted to their host species but not to other potential hosts [38]. Instead, there also records showing that generalist parasites have higher prevalence than specialists in particular host species [27]. According to previous studies, the intensity of infection is correlated with the immune system of the host in different life stages [26] and competition between different parasites in mixed infections [39]. Therefore, the study of seasonal variations in infection intensity is important for us to understand the parasites’ infection mechanism.

In this study we investigate the overall patterns of parasite diversity, prevalence and infection intensity in TIL system with the aim to understand and describe (i) the seasonal trends in infection intensity, (ii) how intensity is affected by the residence types of its hosts and (iii) different seasonal infection patterns between migratory and resident bird species in TIL.

## Methods

### Field sampling and data collection

The research site is located at the Thousand Island Lake (TIL). TIL is situated in an area with limited knowledge about avian malaria diversity and was formed because of construction of a hydroelectric dam in 1959.

**Fig. 1:**
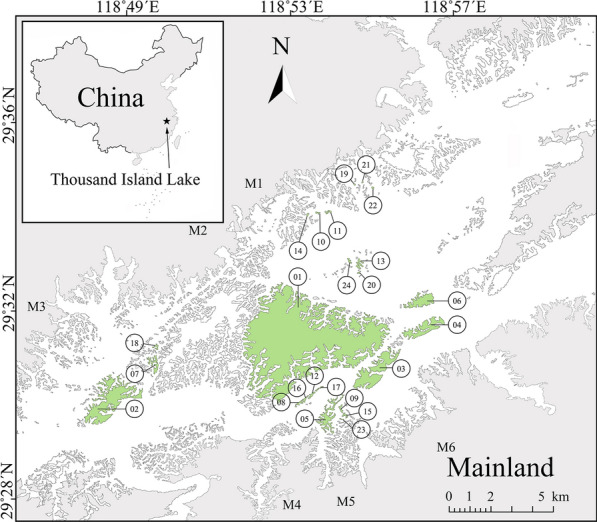
Island distribution in TIL and our sampling locations (islands 1–24, mainland points M1–M6)

Wild birds were captured by mist nets in 2019, 2020 and 2021, starting before their breeding season (April to June) and until their overwinter season (November to January). They were captured at 24 islands and six mainland sampling points (Fig. 1) in Zhejiang Province, China (29°22’–29°50′N and 118°34’–119°15′E).

### Parasite identification, phylogenetic reconstruction and diversity

Blood samples (10 μl–50 μl) were collected from the brachial vein immediately after capture and stored in anhydrous ethanol until DNA extraction. DNA was extracted using a TIAN amp Genomic DNA kit (Tiangen Biotech Ltd., Beijing) following the manufacturer’s protocol and dissolved in 80 μl of TE buffer. Infections were identified using the nested PCR protocol [40], which amplifies a 479‐bp fragment of the *cyt b* gene located in the mitochondrial genome of avian hemosporidians. Positive samples were then sequenced, and the BLAST module in the MalAvi database [41] was used to match the infections to lineages. Each sample was sequenced at least twice to ensure the accuracy of the results. Novel parasite lineages were determined based on any unique one-nucleotide substitution, as recent research shows that just a small difference can cause various ecological characteristics [42]. All the new lineages have been added to the MalAvi database and GeneBank [43].

Positive samples were diluted to 1 ng/μl for further analysis of the infection intensity. However, 59 samples were co-infected and could not be clearly separated, so we removed this part of the data in the analysis of the infection intensity of different parasites.

### Sex determination

The sex of > 50% of bird species cannot be judged directly by external morphology [44]. The individual sex in birds is determined by genes on the two sex chromosomes; males are homogametic (ZZ) and females are heterogametic (ZW). With the development of molecular experiments, the Chromo Helicase DNA-binding gene (CHD gene) on the W chromosome [45] and a very closely related copy of CHD gene on the Z chromosome [46] have been discovered. Since then, several studies have invented primers for sex determination. We used the primers: P2 (5′-TCTGCATCGCTAAATCCTTT-3′) and P8 (5′-CTCCCAAGGATGAGRAAYTG-3′) [44] and 2550F (5′-GTTACTGATTCGTCTACGAGA-3′) and 2718R (5′-ATTGAAATGATCCAGTGCTTG-3′) [47] for the sex identification.

For P2–P8, we started with an incubation step at 94 °C for 1 min, followed by 34 thermal cycles (94 °C for 45 s, 48 °C for 45 s and 72 °C for 45 s) and a final extension step at 72 °C for 5 min. For 2550F-2718R, we started with an incubation step at 94 °C for 5 min, followed by 35 thermal cycles (94 °C for 30 s, 48 °C for 45 s and 72 °C for 45 s) and a final extension step at 72 °C for 7 min.

PCR products were separated in 3% agarose gels, run together with DNA Marker (25–500 bp) in standard TBE buffer and visualized by ethidium bromide staining. We used UV light to observe Z-bands and W-bands. We also checked the results of some of the gender observation results we had recorded when we captured some sex-determining birds. For unclear bands, we performed repeated measurements.

### Intensity quantification

Due to the low intensity often occurring in natural infections, infection intensity is difficult to quantify accurately with blood smears. However, a new method, real‐time quantitative PCR (qPCR), is used to estimate the infection intensities in each individual bird. This protocol has been confirmed to accurately estimate infection intensities compared to microscopic analyses of blood smears [48]. To quantify the total amount of parasite DNA, a general qPCR protocol, which amplifies a fragment of the mitochondrial rRNA from all hemosporidians, was first used [49]. Two pairs of primers were used to amplify the host and parasite DNA fragments, respectively, SFSR/3Fb (5-ACTAGCCCTTTCAGCGTCATGT-3) and SFSR/3Rb (5-CATGCTCGGGAACCAAAGG-3) as host primers, 343F (5-GCTCACGCATCGCTTCT-3), 496R (5-GACCGGTCATTTTCTTTG-3) as parasite primer [50].

All PCRs were performed in an Eppendorf AG 22331 Hamburg qPCR instrument (Germany) using a SYBR‐green reaction kit (Hard-Shell PCR Plates, HSP9655, USA). The thermal profile started with an incubation step at 95 °C for 10 min, followed by 40 thermal cycles (95 °C for 20 s, 60 °C for 20 s and 70 °C for 20 s) and immediately followed by a melting analysis between 47 and 95 °C. Each reaction included 4 ng of DNA template, 0.5 μM of each primer and 5 μl SYBR‐green mix to reach a final volume of 10 μl.

We replicated each sample three times and used the mean value, and we only considered it qualified when the standard deviation of the three values for the same sample was < 0.4; otherwise, the experiment was repeated. At the end of the runs, the amplification curves were checked to obtain threshold cycles (Ct) for each sample with default settings, and results were only accepted if the NTCs did not exhibit fluorescence curves that crossed the threshold line. Melting curves were also inspected to determine false positives (i.e. samples with melting peaks corresponding to non‐specific amplifications).

We used the positive sample 1–1 as the standard to correct differences between each QPCR plate and used it as the golden sample and set the parasite gene concentration and the host gene concentration as 1; we then used it to calculate the concentration of other samples.

$${Q}_{x}={2}^{({Ct}_{gs}-{Ct}_{x})}*{Q}_{gs}$$

Differences in CT values between each PCR plate were calculated and corrected. (When △Ct is > 1, re-experiment)

$${Q}_{n}={2}^{{\Delta Ct}_{parasite}-\Delta {Ct}_{host}}*{Q}_{gs}$$

In our analysis, the logarithm of the infection intensity was taken.

### Phylogenetic reconstruction

We used jModelTest v2.1.1 software [51] to compare the best nucleotide substitution model according to the Akaike information criterion (AIC) and the Bayesian information criterion (BIC) and chose the maximum likelihood (GTR + G + I) model. The maximum credibility tree was selected by TreeAnotator v1.7.5 and visualized in Figtree v1.3.1 (http://tree.bio.ed.ac.uk/software/figtree/). We used the ggtree in R 3.4.3 to groom the phylogenetic tree [52].

### Data analysis

Due to the nonlinear correlation between parasite and time, we used the generalized additive model (GAM) to analyze the trend of infection intensity by month. GAM is a generalized linear model offering a middle ground that can be fit to complex, nonlinear relationships and make good predictions in these cases [53]; it has been used to examine nonlinear relationships between infection status and seasonal changes in studies [19, 35, 54, 55]. By generalized cross-validation, smoothing parameters were automatically selected by penalized likelihood maximization, retaining more parsimonious linear function and penalising more complex nonlinear functions. Therefore, we established the generalized additive model in R package mgcv, with date as the independent variable and infection intensity as the response variable. We used binomial errors and a logit link to incorporate the smoothed function of sampling date as a model predictor. Generalized cross validation (GCV) was used to choose the number of iterations for smoothing parameter selection. To exclude hazardous results from our analysis, we tried to remove parasites that only appeared once and then compared the experimental results with the results of all parasites. We found that this did not affect the trend (Additional file 1: Fig. S1). Since there was many new lineages of *Leucocytozoon*, removing this part of the data would only have been partial, so we finally decided to keep it. We also analyzed the monthly trends of infection intensity of resident birds, summer visitors and winter visitors, and separated trends into females and males, also in the different genera, *Plasmodium*, *Haemoproteus* and *Leucocytozoon*.

## Results

**Fig. 2:**
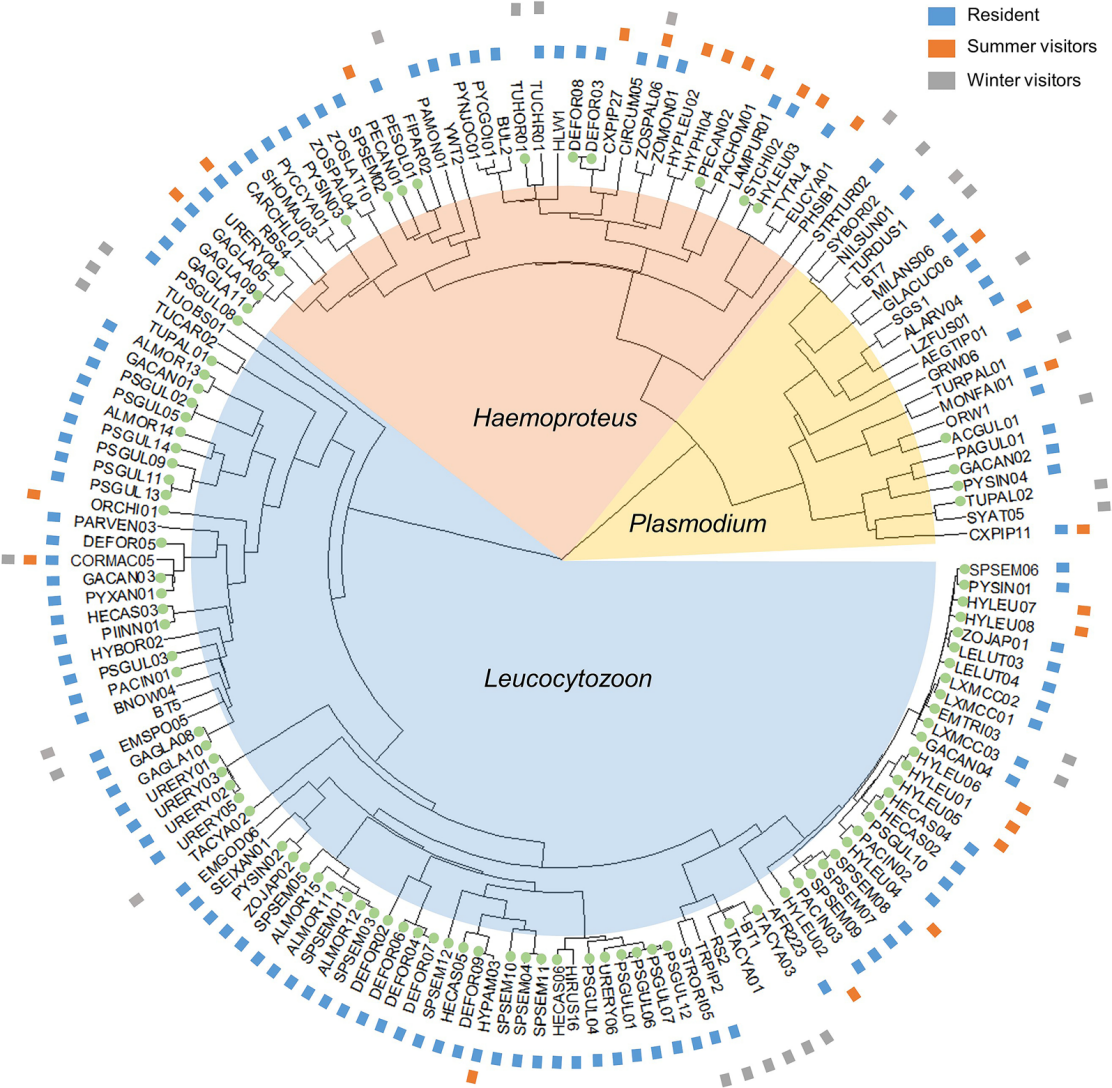
Phylogenetic trees for all the species/lineages found in TIL. Green points represent new lineages

We surveyed parasite infections of blood samples from a total of 1101 individuals from 86 avian host species on 24 islands and 6 mainland areas. We detected 95 lineages of *Leucocytozoon* in 206 individuals, 21 lineages of *Plasmodium* in 108 individuals and 40 lineages of *Haemoproteus* in 150 individuals. The total prevalence was 37.0% (407 individuals) (Additional file 1: Table S1), which included 59 samples co-infected. Due to the survey of avian blood parasites in Asia at the initial stage, we found 95 new lineages, accounting for 60.9% of the total lineages; most new lineages occurred in *Leucocytozoon* genus (Fig. 2).

### Seasonal trend of infection intensity

**Fig. 3:**
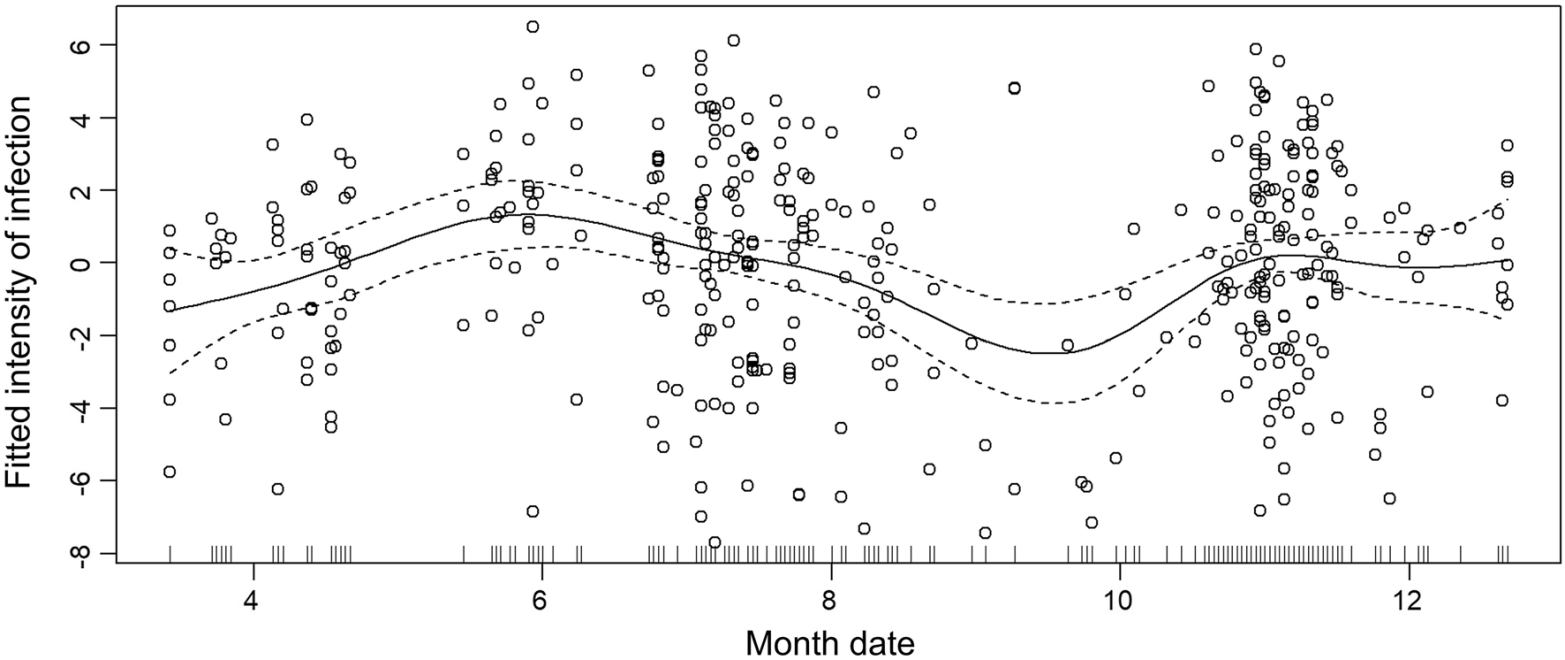
Monthly trends of infection intensity for all parasite-infected passerine bird samples

**Fig. 4:**
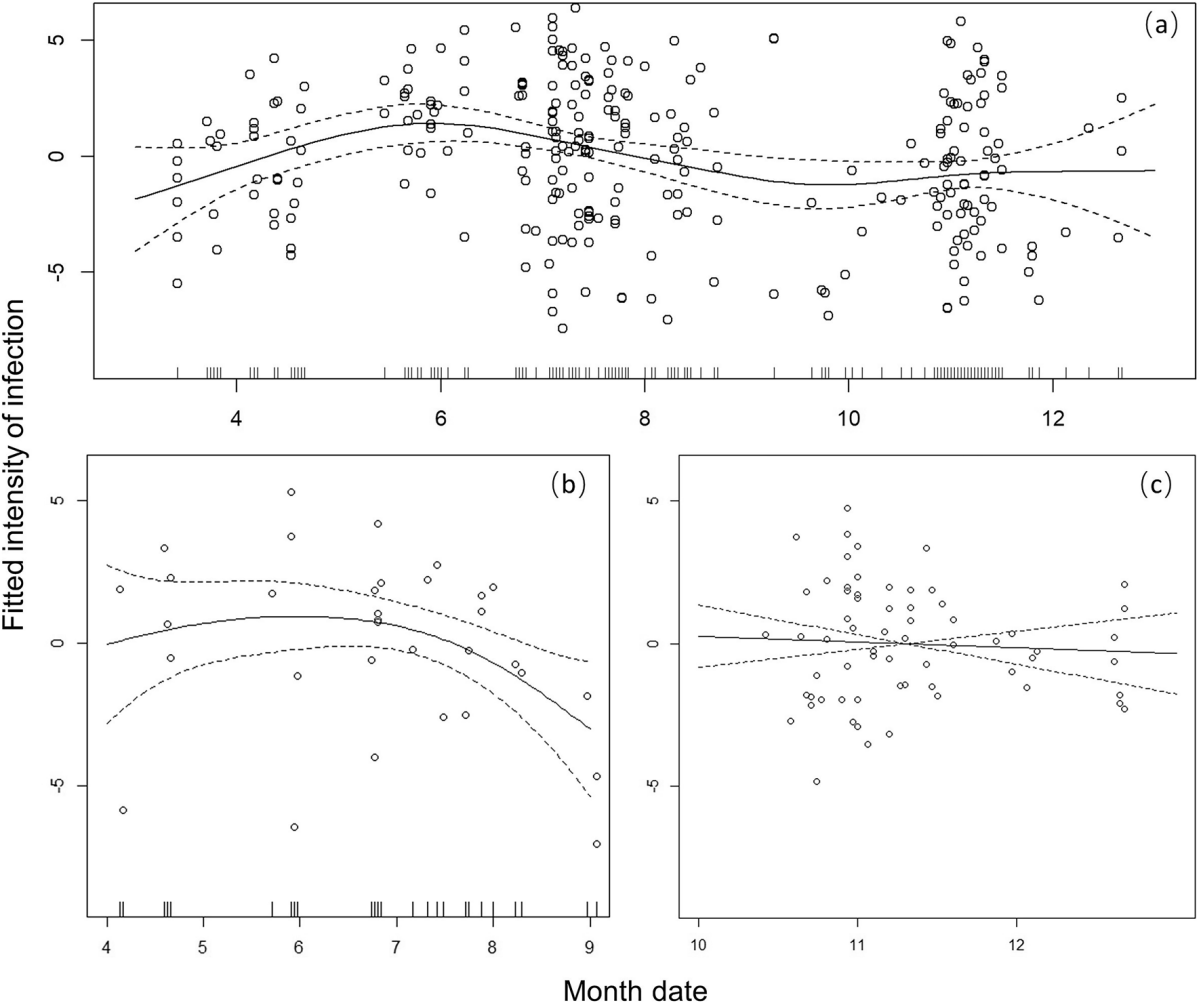
Monthly trends of infection intensity for **a** resident, **b** summer visitors, **c** winter visitors

GAM examining seasonal variation in the infection intensity. The curves represent the estimated model effect as predicted by date and the fitted infection intensity for overall infections. We found significant variation in infection intensity with date (ANOVA, *F*_(8.33)_ = 3.10, *P* = 0.003; Fig. 3). The average infection intensity during the breeding seasons (April to June) and overwinter seasons (October to January) has a growing trend and declines from June to October. The result is consistent with seasonal variations of infection rates in our other unpublished study (Wu et al. Unpublished Data). Moreover, the trend of resident birds has the same shape as our total data, but the relative changes tend to be flat (ANOVA, *F*_(5.26)_ = 3.78, *P* = 0.002; Fig. 4a). For the summer visitors, the infection intensity decreases gradually (ANOVA, *F*_(2.61)_ = 2.76, *P* = 0.084; Fig. 4b), while the infection intensity for winter visitors is continuously high with only a slow, non-statistically significant decline in the overwinter season (ANOVA, *F*_(1)_ = 0.23, *P* = 0.634; Fig. 4c).

### Different trends of infection intensity in females and males

**Fig. 5:**
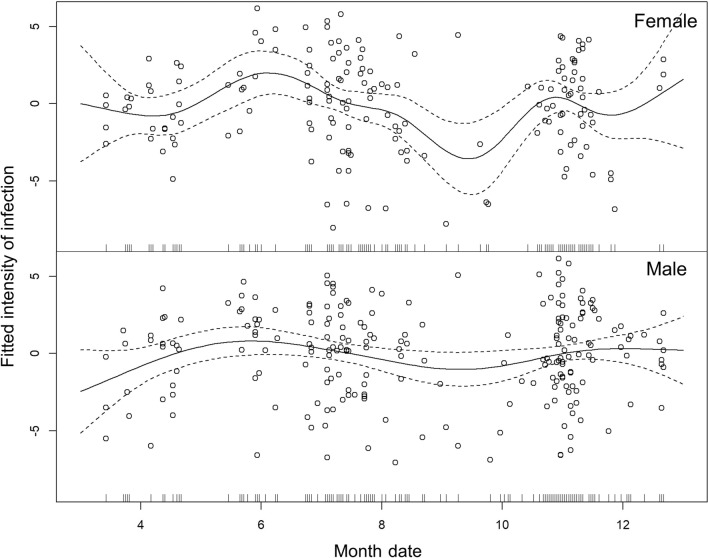
Monthly trends of infection intensity in females and males

In the individuals we collected, 39.3% of females (175 out of 445) were infected, which is higher than the 35.4% in males (232 out of 656), but prevalence between females and males did not show any significant difference (Chi-square test*χ*^2^ = 1.78, *df* = 1, *P* = 0.250). Instead, the infection intensity in females is higher than in males (t-test, *t*_(354.6)_ = 2.00, *P* = 0.047). By comparing the monthly trends of infection intensity, we can observe a sharp trend in females (ANOVA, *F*_(8.72)_ = 1.94, *P* = 0.034; Fig. 5). Instead, the trend in males does not show significant variation (ANOVA, *F*_(5.40)_ = 1.50, *P* = 0.195; Fig. 5).

### Different trends of infection intensity in various genera of parasites

**Fig. 6:**
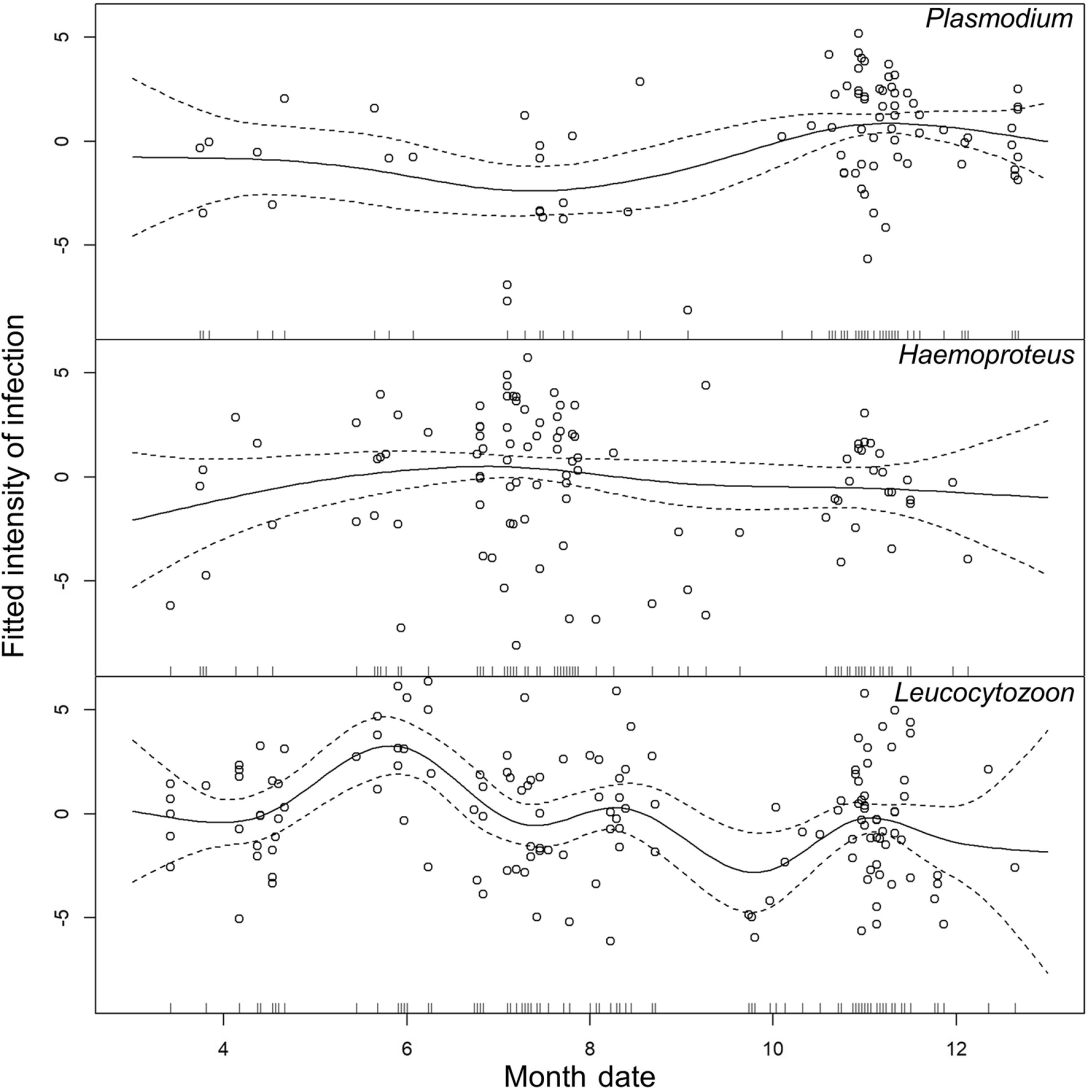
Monthly trends of infection intensity in *Plasmodium*, *Haemoproteus* and *Leucocytozoon*

The three different parasite genera have various trends and high points in different seasons (Fig. 6). As described in Methods, we removed the 59 co-infection samples for analysis to ensure the accuracy of infection intensity in each genus. For the genus *Leucocytozoon*, the trend of infection intensity reached its highest point in June (ANOVA, *F*_(8.59)_ = 3.98, *P* < 0.001; Fig. 2). It is also likely due to the fact that the main hosts of *Leucocytozoon* might be resident birds (Table 1). The infection of the genus *Plasmodium*, concentrated in winter, is at a high level of intensity, which is also consistent with our results that *Plasmodium* infection is highly prevalent in winter visitors (ANOVA, *F*_(4.43)_ = 4.68, *P* = 0.001; Table 1; Fig. 6). In addition, the trend of infection intensity of the genus *Haemoproteus* does not show a significant variation (ANOVA, *F*_(3.34)_ = 1.17, *P* = 0.324; Fig. 6).

**Table 1:** Prevalence of infection in *Plasmodium*, *Haemoproteus* and *Leucocytozoon* in different types of residential birds

Parasite genera	Resident birds (%)	Summer visitors (%)	Winter visitors (%)
*Plasmodium*	5.6	3.4	34.1
*Haemoproteus*	13.2	17.0	12.9
*Leucocytozoon*	20.4	13.6	17.1

## Discussion

How parasite intensities differ based on season or migratory behavior of its host is important in order to understand the mechanisms of transmission of parasites and diseases. By studying the bird community in TIL, we have shown that there are peaks of infection intensity during the summer. Correspondingly, Huang et al.'s recent research of a wild bird community in southern Sweden also found peaks of infection intensity and prevalence in summer, which can be explained by spring relapse [56]; Šujanová et al. found the infection intensity of a local avian community in Slovakia peaked during summer around mid-July [57]. From the perspective of prevalence, Schrader et al. found that Red-bellied woodpeckers (*Melanerpes carolinus*) have a peak of prevalence during summer [21]. The seasonal trend between intensity and prevalence in TIL coincides with an earlier study [58], which shows a positive correlation between prevalence and infection intensity. Our other unpublished research (Wu et al. Unpublished Data), which focuses on the trend of prevalence, also shows a similar seasonal trend. All these studies consistently show that infection intensity peaks during summer; our study supports these findings.

Some avian hemosporidian parasites might become dormant during the winter, followed by a relapsing of parasites during spring, a so-called spring relapse [24]. One reason that we observe that the intensity has a peak during summer can be explained by possible spring relapses, which cause higher infection intensity and prevalence [55, 56, 59]. An alternative or additional explanation of the high intensity during the spring and breeding period of the birds might be related to the immunity of birds [56, 60]. In the present study, we observed that the intensity in females is indeed higher than in males (*P* = 0.024), which may also be caused by the difference in immune investments between females and males [61]. According to the findings of Šujanová et al., infection intensity tended to be higher in older birds [57]. Considering supressed immunity activity in reproductively active birds [56], we guess the supressed immunity may also differ between sexes. The seasonality trends of avian immunity between different sexes would also be an interesting area to study in the future and could better explain variations in infection intensity during the breeding season.

Impact of migration on local species could help us to better understand transmission of parasites and diseases [28]. Notably, in our study, summer visitors have a high infection intensity during the initial stage of arrival at TIL but show a downward trend afterwards (Fig. 4). The avian migration study in Beijing shows that parasites can be transmitted during migration. Migratory birds can be infected at the breeding ground [62]. Migratory birds are often exposed to different parasite fauna during their migratory cycle, so they have more chances of being infected with different lineages than residents, which causes the interspecific differences in investment in immune defense [63]. There are also studies testing metabolic rates and exercise endurance in great reed warblers (*Acrocephalus arundinaceus*), showing that phenotypic changes associated with preparation for migration are similarly unaffected by parasitemia [64]. Another study also shows that the physiology of migratory birds after migration reduces parasite survival [65], which could be a reason for the reduction of infection intensity during the summer. In our study, both summer visitors and resident birds show a reduction in parasite intensity after the breeding season.

Parasites play an important part in bird migration [66]. The recent study of the hemosporidian infections of wild birds belonging to the order Columbiformes in the Northern Hemisphere also found a higher parasite prevalence in long-distance migratory birds [67]. However, contrary to overwintering migratory birds, which are rarely infected with hemosporidian parasites as reported in previous studies in Hispaniola [65], in our research, winter visitors have a significantly higher prevalence (Chi-square test, *χ*^2^ = 477.4, *df* = 1, *P* < 0.001) and infection intensity (t-test, *t*_(202.1)_ = 3.75, *P* < 0.001) than residents. The reason for this difference could be the extremely high rate of *Plasmodium* infection (Table 1) in migratory compared to resident birds. Moreover, we found that the most prevalent *Plasmodium* SYAT05 (46 out of 108 individuals infected by *Plasmodium*) only exists in winter visitors and some passing birds but never infect residents in TIL. Therefore, we believe that the winter visitors are infected with *Plasmodium* in the northern living area or along the way before arriving in TIL and rarely spread it to local resident birds. Furthermore, we found that, in the winter visitors, the intensity during the winter continues at a high infection intensity and does not show significant changes (Fig. 4c), which is different from our results in summer visitors. The infection patterns of summer and winter visitors may be different and should be studied separately in follow-up studies.

The monthly intensity trends in different parasite genera also show different patterns (Fig. 6). *Plasmodium* infection has a high prevalence and maintains a high infection intensity during the winter; *Haemoproteus* mainly infects birds in July and November, and the intensity does not obviously change over the years; the seasonal trend of *Leucocytozoon* is consistent with the overall trend. Moreover, since the infection intensity of *Leucocytozoon* is lower than for *Plasmodium* (*t*-test, *t*_(171.8)_ = 3.75, *P* < 0.001) and *Haemoproteus* (t-test, *t*_(207.8)_ = 3.45, *P* < 0.001), we can infer the following reasons. First, the dynamics of the infections are totally different so it is difficult to compare between genera. Different species of parasites infect different blood cells. Since *Plasmodium* have asexual reproduction in the blood [68], they will have more copy numbers of parasites in qPCR. Then, due to the extremely high variation and diversity of *Leucocytozoon* in nature [69], there are 82.1% new species that are not found in MalAvi yet [41], so the adaptability to the host might be low.

Moreover, the infection by parasites can damage organs [70] and cause some wild birds to have limited mobility or die, which can lead us to underestimate the infection intensity and prevalence. These problems have also appeared in previous studies [58, 71]. The solution to this problem needs to be tackled by follow-up infection experiments and investigation of mosquitoes and blackflies [72]. In addition, different species of parasites have different vectors and host preferences [73]. Seasonal trends are also found in the vectors which are affected by precipitation [74]. These factors also account for seasonal variations in different species. The collection of mosquitoes will help us understand the transmission mechanism of different parasites, which will also be part of our future studies.

Our research shows the total infection intensity variations among different resident birds and different seasons in TIL. However, habitat fragmentation also has an impact on parasite infection [75, 76]. Anthropogenic habitat change, like deforestation, can affect host-parasite systems and reduce the rate by which parasitic infection are accumulated in the populations [77]. The prevalence of *Plasmodium* infections showed significantly higher levels in undisturbed forests [78]. Island systems may act as the effective natural laboratories for biodiversity, playing an important role in the ecology and disease ecology studies [79, 80]. TIL, as an ideal fragmentation experimental base, has clear geographic boundaries and the same formation history and isolation medium (water). Due to their short formation time, they were not affected by historical evolutionary processes and geological events, which make it easier to reveal complex ecological mechanisms. The prevalence and intensity between different islands, which could help us better analyze the impact of habitat fragmentation on blood parasites, will also be examined in our future studies. By collecting data over a long time period, we may find changes in infection due to vectors’ change, and perhaps *Plasmodium* will also infect residents in future studies, after better adaptation to the hosts and environment. Therefore, long-term investigation is very important to elucidate this ecological mechanism.

Finally, there are still some limitations of our study. First, morphological species could not be identified without blood smears, so we can only explain that there were new lineages instead of new species. This limitation may also make us underestimate some co-infections between *Plasmodium* and *Haemoproteus*. Hence, using different sets of primers in the future to study multiple infections [81] may solve this problem. Second, for the measurement of infection intensity, because of the lack of participation of standard infection intensity obtained from blood cells count, all our intensity values became a relative value. Meanwhile, the cells infected by *Plasmodium* and *Haemoproteus* were different from those infected by *Leucocytozoon*, so we could not completely compare the intensities between different parasite genera.

## Conclusions

Our study shows the distribution and seasonal variation intensity trend of blood parasites in TIL. Most of the new species are of the *Leucocytozoon* genus. The seasonal variation has peaks at the beginning of summer and winter. A similar trend is also shown in the *Leucocytozoon* genus. The differences between migratory birds and residents have also been discussed. The infection intensity of summer visitors gradually decreases after they arrive at TIL. However, winter visitors have a constant high intensity during the winter. They are almost always infected by *Plasmodium* spp. and rarely share parasites with resident birds. We observe a significant seasonal trend of infection intensity in females but not in males. Different parasite genera shows different monthly trends of infection intensity. Our subsequent investigation will focus on vectors and fragmentation to study the transmission mechanism of parasites and diseases after reaching TIL by longer-term sampling of mosquitos and bird blood samples.

## Supplementary Information

**Additional file 1: Table S1.** The list of birds we collected at TIL and their residence types and prevalence of different parasite species. **Figure S1.** The monthly trends of infection intensity for parasite-infected passerine bird samples except where the parasite species only appeared once.

## Data Availability

All the linages have been uploaded to GeneBank (number OQ745964-OQ746058).

## Abbreviations

qPCR: Quantitative PCR
TIL: Thousand Island Lake
cytb: Cytochrome b
GAM: Generalized additive model
CHD gene: Chromo helicase DNA-binding gene
